# Focus on current and future management possibilities in inflammatory bowel disease-related chronic pain

**DOI:** 10.1007/s00384-018-3218-0

**Published:** 2018-12-19

**Authors:** Anna Zielińska, Maciej Sałaga, Marcin Włodarczyk, Jakub Fichna

**Affiliations:** 1 Department of Biochemistry, Faculty of Medicine, Medical University of Lodz, Mazowiecka 6/8, 92-215 Lodz, Poland; 2 Department of General and Colorectal Surgery, Faculty of Military Medicine, Medical University of Lodz, Lodz, Poland

**Keywords:** Inflammatory bowel disease, Chronic abdominal pain, Nociception, Pain managemant, Pharmacotherapy

## Abstract

**Introduction:**

Visceral pain is a symptom reported by over 70% of inflammatory bowel disease (IBD) sufferers. So far, a single, specific cause of this debilitating state has not been established. Chronic pain is one of the most important factors decreasing the quality of life in IBD course. Concurrently, management of pain is the most challenging issue encountered by clinicians in IBD treatment.

**Areas covered:**

This review focuses on pathophysiology of inflammatory bowel disease-caused visceral pain and explores currently available approaches to its management. We also covered recent pharmacological developments in the field.

**Conclusions:**

Pain-related disability has major effects on quality of life and on functional and social outcomes in IBD patients. Currently, there is no one standardized method of managing chronic visceral pain in IBD. Therefore, future development, focusing primarily on alleviating the pain, but also on reducing inflammation, is essential.

## Chronic abdominal pain in inflammatory bowel diseases

Inflammatory bowel diseases (IBDs), including Crohn’s disease (CD) and ulcerative colitis (UC), are chronic inflammatory disorders with periods of exacerbation and remission which predominantly affect the gastrointestinal (GI) tract. IBD symptomatology is non-specific and very diverse. General symptoms such as fever, weakness, and weight loss are accompanied by intestinal symptoms associated with chronic inflammation of the intestinal mucosa, such as abdominal pain and chronic diarrhea [1]. Chronic abdominal pain is a commonly experienced and debilitating symptom of IBD, with up to 70% of patients experiencing pain in exacerbation of disease. Pain in IBD patients may occur during the course of the disease secondary to acute inflammation, strictures, adhesions, bowel obstruction, bowel dysmotility, intestinal fistulas, and/or abscess formation [2, 3]. Reducing abdominal pain is the main therapeutic target for IBD therapy; however, pain severity does not always correlate with endoscopic and clinical activity of disease, and a significant number of patients (20-50%) report to continue experiencing chronic pain during periods of remission [4].

### Pathogenesis of pain in inflammatory bowel diseases

The pathogenesis of pain in IBD patients remains unclear, but several potential pathological mechanisms, including inflammation, bowel obstruction, psychological, psychosocial, neurobiological, and genetic factors, have been suggested [5]. In recent studies, poor correlations between chronic abdominal pain intensity and IBD activity indices were observed, what emphasizes the multifactorial nature of pain in IBD [6, 7].

The pain in IBD patients is related to stimulation by pain-producing or nociceptive factors of specialized primary afferent neurons called nociceptors. Membrane receptors on nociceptors are responsive to different stimulus modalities, including chemical, thermal, and/or mechanical [8, 9]. The nociceptive transmission from the primary afferents to the second-order neurons in the spinal cord proceeds through excitatory glutamatergic synapses. Neural impulse is further transmitted, along the spinal cord, to the brainstem, the thalamus, and consequently to higher centers of the brain. Sensation of pain arrives when the neural signal reaches specific parts of the cerebral cortex, including the somatosensory cortex, insula, and anterior cingulate cortex [10]. Multiple centers within the brain are able to modulate the perception and response to nociceptive stimuli that ultimately lead to the experience of chronic pain [11].

Recurrent inflammatory process and release of mucosal signaling molecules (e.g., nerve growth factor, glial cell-lined derived neurotrophic factor) as well as changes in ion channel expression (TRPV1/TRPA1) in chronic IBD-related inflammatory intestinal lesions can result in development of visceral hypersensitivity and chronic visceral pain. Of note, modulation of synaptic communication between nociceptors and second-order neurons in the spinal cord in non-inflammatory conditions may also lead to hyperalgesia and secondary persistent pain bowel pain [12]. Recurrent visceral nociceptive stimulation may activate the *N*-methyl-D-aspartate receptors and influx of calcium in higher/second-order sensory neurons, resulting in long-lasting neuronal excitability in the absence of inflammatory process [13]. In addition, activation of structures within the brainstem, specifically the periaqueductal gray matter of the pons and the rostroventromedial medulla, can either inhibit or facilitate the incoming transmission of sensory information, resulting in chronic abdominal pain [14].

Central processing within the brain, such as psychological stress and excitement, may also have a role in hyperalgesia and development of chronic IBD pain, for example via disturbing the mechanisms in the brain-gut axis. Stress-induced alterations in gastrointestinal inflammation may be mediated through changes in the hypothalamic-pituitary-adrenal (HPA) axis function and alterations in bacterial-mucosal interactions as well as via mucosal mast cells and mediators such as corticotrophin releasing factor (CRF) and thereby the release of circulating inflammatory cytokines (eg IL-6) [15, 16].

Emotional and cognitive processes in prefrontal cortex may also lead to hyperalgesia and secondary persistent bowel pain by modulating descending inhibitory signals [17].

All of these modulatory mechanisms have been implicated in chronic pain conditions. There are several pathways in which pain from visceral organs is unique compared to somatic pain. The majority of visceral primary afferents are polymodal—capable of sensing mechanical, thermal, and chemical stimuli which permits them to transmit a diversity of signals from the environment [18]. Most of these sensory afferents are also able to respond to graded stimuli well into the noxious range; in a sense, almost all visceral sensory afferents are capable of generating pain. Visceral organs have a dual nerve supply, and both sets of nerves may possess distinct functional functions and contribute in different way to visceral pain [19]. Once in the spinal cord, the visceral afferents arborize into an extensive network that extends throughout the spinal cord and overlaps with other primary afferent neurons [20]. Consequently, visceral stimuli, including nociceptive ones, are capable of recruiting large sections of the central nervous system (CNS), resulting in the diffuse, poorly localized, and often referred nature of visceral pain [21]. Chronic abdominal pain in IBD patients is a complex phenomenon driven by a range of peripheral and CNS processes.

## Management of pain in inflammatory bowel diseases

Given the complex interplay between multiple factors leading to chronic abdominal pain and its significant consequences, effective management is critical in IBD patients. Current treatments for pain management in IBD carry a number of risks and limitations. When pain is associated with exacerbation of IBD, the primary treatment will often be intensification of IBD therapy. However, pain may persist despite adequate IBD therapy, or pain may arise from a non-IBD source. Escalating current pharmacotherapy or exploratory surgery for pain in the absence of inflammatory markers or intestinal lesions can have iatrogenic side effects, potentially exacerbating disease activity, psychological distress, and worsening patient’s quality of life (QoL). Pain management with antispasmodics, anticonvulsants, tricyclic antidepressants, and cyclooxygenase-2 (COX-2) inhibitors may provide pain relief, yet their long-term use can exacerbate gut symptoms and bowel dysmotility [22]. Of note, a significant number of patients use opioids or marijuana for pain control despite psychological and disease-related risks [23, 24].

Importantly, patients with unrelenting abdominal pain are some of the most challenging patients for practitioners to manage. When abdominal pain occurs in the absence of inflammation, it can lead to multiple provider visits and potential patient frustration that their complaint is not being given careful attention (Table 1).

**Table 1:** ᅟ

Current management of IBD pain	
NSAIDs	• Mainly used in extraintestinal manifestations • Inhibition of prostaglandin production by COX enzymes • May exacerbate the course of the disease in some subgroups
Opioids	• Pain relief and anesthesia • Immunosuppressive effect • Higher exacerbation, surgical interventions and mortality rate • Severe side effects (including narcotic bowel syndrome) • High risk of developing dependency
Antidepressants	• Use in anxiety and depression in IBD • Adjuvant analgesic, reduction of chronic opioid use • Lack of randomized trials describing efficacy in pain mitigation
Anticonvulsants	• Use in neuropathic and visceral pain • Beneficial effect on visceral hypersensitivity
Psychotherapy	• Complementary therapy, useful especially in chronic opioid therapy and in patients unresponding to medical interventions • Proved benefit in increasing QoL

### Non-steroidal anti-inflammatory drugs

In IBD patients, non-steroidal anti-inflammatory drugs (NSAIDs) are mainly used in the management of extraintestinal manifestations of IBD such as spondyloarthritis. NSAID use is highly effective in other inflammatory arthropathies where it is often recommended as a first-line therapy [25, 26]. NSAIDs act as anti-inflammatory analgesics primarily by inhibiting the production of prostaglandins by cyclooxygenase (COX) enzymes. Under conditions of inflammation, NSAIDs’ analgesic effect is mediated through inhibition of the inducible form of the enzyme, COX-2. Non-selective NSAIDs also inhibit the constitutively produced COX-1 enzyme, which maintains the mucosal integrity of the intestine. The combination of reduced prostaglandin production and direct toxicity results in the inflammatory intestinal lesions as side effects associated with prolonged use of NSAIDs [27, 28].

In contrary to the generally accepted belief, a consistent association between acetaminophen, NSAIDs, or COX-2 inhibitor use and risk of CD and UC exacerbation was not confirmed. It was showed that IBD is a heterogeneous disease, and therefore, there may be subgroups of patients particularly at risk of disease exacerbation with use of NSAIDs [29]. Given the limited data, avoiding the use of NSAIDs in most cases would be prudent. In patients with debilitating arthritis that cannot be controlled by other means, cautious use of selective COX-2 inhibitors could be considered [30]. However, safety data on regular, long-term use of these medications in patients with IBD is lacking. Therefore, future studies focused on examining predictors of disease exacerbations/progression with use of NSAIDs are needed to clarify this controversial aspect of pain management.

### Opioids

Opioids are substances, both natural and synthetic, which act on opioid receptors to produce morphine-like effects [31]. Medically, they are primarily used for pain relief, including anesthesia, as they bind to receptors associated with pain, reward, and addictive behaviors. They are used by healthcare providers to relieve pain that cannot be diminished with less powerful drugs [32]. However, the role of these drugs in chronic non-cancer pain (CNCP) involves several problematic issues, including side effects, abuse, and diversion to other individuals. Alarming is that despite the above-mentioned concerns, the prolonged use of opioid therapy for CNCP has been increasing dramatically [33, 34]. While evidence shows pain relief with short-term opioid use, this benefit often does not translate into improved functioning over the long term. The few studies of opioid use for CNCP have shown small-to-no benefit, resulting in some disillusionment with long-term opioid therapy [35, 36].

There is surprisingly limited number of information about the prevalence of opioid use in IBD. An early study reported chronic opioid use in 30% of IBD patients, although this study only included patients referred for psychiatric evaluation [37]. In all patients presenting to an IBD clinic, the frequency of opioid use ranged from 3 to 13% [38, 39]. Opioid use may be a marker for more severe IBD course; consequently, it was found that patients who were treated with opiates are related with higher exacerbation rate of disease and pain and were almost twice as likely to require surgical intervention [40, 41]. Interestingly, over half of IBD patients who returned for follow-up care were able to discontinue opiates [40]. These patients were more likely to be adherent to medical therapy and to have disease activity and pain under control [42]. This finding suggests that most patients with IBD can be successfully prevented from opiates if their IBD remission and pain relief is achieved with alternative treatment.

In a recent study, it was observed that around 5% of individuals with IBD become heavy users of opioids over the course of the disease, and that heavy opioid use is more common in women. Women with IBD are also using opioids at higher rates than their age-matched controls for up to 5 years before their diagnosis of IBD, possibly suggesting a delay in diagnosis of IBD among women [43]. The use of opioids at low intensity before diagnosis is strongly associated with heavy use of opioids later in the course of disease, and the risk is also increased for those diagnosed at a young age [39].

The prolonged use of opiates was found to be associated with increased mortality rate among IBD patients, although this association was not significant when the analysis adjusted for other risk factors. Other opiate side effects such as nausea, respiratory depression, sedation, and euphoria/dysphoria are common but will usually subside with time [44]. Constipation is an exception to this statement, and routine initiation of a bowel regimen is recommended when starting opiates [45]. While constipation would be a welcome respite for most IBD patients, it could be dangerous if patients are at risk for developing toxic megacolon [46]. Narcotic bowel syndrome is another particularly significant clinical complication of prolonged opioid use, and it refers to a spectrum of disorders that develop secondary to the actions of opioids on the GI tract and the central nervous system. As opiate use has increased, so has the recognition that these agents have a number of adverse effects on the GI tract. Prolonged opiate use may cause in same patients neuroplastic changes that result in bowel hyperalgesia and tolerance enhancement [47]. In recent studies, at least three recognized mechanisms leading to enhancement of pain with the prolonged use of opioids have been suggested: the existence of a bimodal opioid regulation system where preferential activation of excitatory pathways over time may lead to opiate tolerance and pain intensification, counter-regulatory systems, with release of anti-opioid neuromodulators such as dynorphin and cholecystokinin that oppose opioid antinociceptive function, and glial cell activation that produces morphine tolerance and increases opiate prompted pain [41].

The opiate use was also a risk factor for serious infections, even after adjusting for severity of disease and use of immunosuppressive agents [48]. In a recent study, it was suggested that opiates may be masking early signs and symptoms of infection. However, another possibility is that opiates have a direct effect on infection due to decreased gut motility and bacterial translocation through the damaged mucosa of the inflamed intestine [49]. There is also evidence for opiates having direct immunosuppressive effects that could predispose patients to infections [50]. Other major concerns when prescribing opiates are addiction and diversion [51].

Strategies directed at limiting and closely monitoring the use of opioids in IBD patients may serve to reduce the risk of subsequent prolonged opioid use. Patients deemed to be at high risk for abuse of prolonged opiate therapy may necessitate careful psychological evaluation and close monitoring by physicians. Patients who have the most difficulties with opioid therapy are often clinically depressed and need urgent psychological support [52].

### Antidepressants

Antidepressants are commonly used to treat symptoms of anxiety and depression in IBD. In the management of pain in IBD, antidepressants are suggested as an adjuvant analgesic to reduce the need for chronic opioid therapy [53]. However, there have been only few studies in IBD patients. The study of paroxetine showed significant improvement in several components of QoL, although this study only included patients who had already been diagnosed with depression [54]. The studies indicate that antidepressants may have a beneficial effect on IBD course, but it is currently not possible to determine their efficacy on pain in IBD patients because of the lack of randomized trials [55].

### Anticonvulsants

Anticonvulsants also commonly known as antiepileptic or as antiseizure drugs are a diverse group of pharmacological agents mainly used in the treatment of epileptic seizures and neuropathic pain. More recently, anticonvulsants, predominately gabapentin and pregabalin, have been used to treat visceral pain [56]. Recent studies of gabapentin and pregabalin have shown beneficial effects on visceral hypersensitivity [57, 58]. However, further clinical trials are required to confirm the role of anticonvulsants in management of visceral pain in IBD patients.

### Psychotherapy

Nowadays, international guidelines by the European Crohn’s and Colitis Organization (ECCO) suggest psychotherapy and psycho-pharmacological treatments as playing an important role in IBD care. Psychotherapy can be an effective complementary therapy to help patients cope with pain that cannot be entirely eliminated. Some form of psychotherapeutic intervention is routinely recommended when using chronic opioid therapy [59]. Recent meta-analyses have shown significant benefits with cognitive behavioral therapy and psychological support, especially in IBD patients who have not responded to medical therapy [60, 61].

In recent systematic review, promising findings for psychosocial interventions, including stress management techniques and coping skills training in management of chronic abdominal pain in IBD patients, were observed [62]. Recently application of a psychosocial intervention, alongside IBD medication, for pain management was recommended. In particular, the consistent association identified between depression and anxiety and pain suggests that treatment of mood-related issues may improve pain levels and pain-related QoL. Additionally, targeting pain-specific thoughts and behaviors such as pain catastrophising and fear avoidance may in turn show beneficial effects on mood as well as pain [63]. It was also suggested that the pain acceptance and resilience/psychological well-being may be useful target for an intervention in buffering the impact of pain on patients with IBD [64].

## Recommendations of pain management in IBD patients

**Fig. 1:**
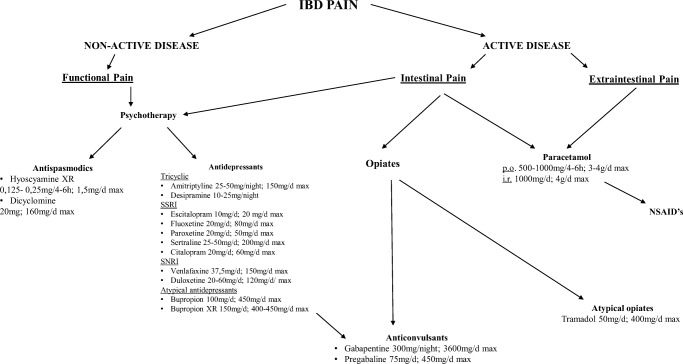
Current state of art- advised treatment od chronic abdominal pain in IBD patients

Currently, there are no specific therapies for treating visceral pain in IBD patients. Consequently, a multidisciplinary approach similar to that used for patients with other chronic pain conditions should be recommended. This approach should involve a combination of non-invasive measures such as aerobic exercise, physical therapy, medications, and psychotherapy (Fig. 1). Such approach has been shown to have positive outcomes for management of chronic pain in IBD patients [65]. Suggested treatment algorithm of pain management in IBD should always involve clinical evaluation of the patient for active disease. If escalation of IBD therapy does not relieve symptoms, then over-the-counter analgesics may be initiated. Care should be taken to rule out complications such as strictures or adhesions that will respond only to surgical treatment. Given that anxiety and depression are frequent comorbidities of IBD, formal psychiatric evaluation and treatment can be helpful. The use of an antidepressant, especially tricyclic antidepressants, may help with pain even in the absence of a psychiatric diagnosis. Opiates should be used with caution in IBD patients, preferably for a well-defined interval such as during induction of remission or during the postoperative period.

## Future perspectives of pain treatment in IBD

Abdominal pain is often described by IBD patients as cramping sensation varying in intensity. There are two types of abdominal pain—somatic, which is of musculoskeletal origin and visceral, which in IBD is caused by widely distributed inflammation or intestinal obstruction. Inflammation is associated with alterations in certain gene expression in neurons as well as immune cells. The changes in intrinsic sensory neuron properties and in gene expression regulation of nociceptive specific proteins lead to sensitization of primary afferent neurons. That contributes to increased production of pro-inflammatory molecules which are involved in painful tissue damage, swelling, edema, and vasodilation [66].

Development of novel analgesics for IBD therapy focuses primarily on finding new molecular targets and/or designing novel molecules with higher efficacy and improved safety profile. Another useful strategy may be to simultaneously act on several targets to alleviate pain and reduce inflammation in order to prolong the therapeutic effect.

One of the early strategies proposed for the treatment of pain in IBD was targeting *N*-methyl-D-aspartic acid (NMDA) receptors that occur in the enteric nervous system [67]. It has been found that these receptors are upregulated in the myenteric plexus of the colon up to 28 days after exposure to pro-inflammatory agent trinitrobenzene sulfonic acid (TNBS). Zhou et al. have reported that expression of certain splice variants of NR1 subunit of NMDA is enhanced in the spinal cord of hypersensitive rats treated with TNBS [68]. These results suggest that colonic myenteric plexus NMDA receptors contribute to the visceral hypersensitivity in the colon [69]. Moreover, it has been shown by the same group that serine phosphorylation of the NR1 appears 14 days after TNBS in contrast, to the control group where no NR1 expression was observed. These results suggest a role for colonic-NMDA receptor phosphorylation in the development of neuronal plasticity following colonic inflammation, and NR1 phosphorylation may partially explain visceral hypersensitivity during colitis [70]. In summary, those findings suggest the potential use of NMDA antagonists, such as dextromethorphan or ketamine in the treatment of visceral pain. However, none from this class of drugs has proved successful in the IBD setting.

One of the recent advances in the field of pharmacological treatment of pain is targeting nociception receptors (NOPs) in the gut. Nociceptin is an endogenous, bioactive heptapeptide, which is a product of an enzymatic degradation of its precursor: prepro-nociceptin [71, 72]. In the GI tract, NOPs are expressed on muscle cell membranes and neurons, as well as the immune cells that infiltrate the mucosa. It was showed that nociceptin is involved in the regulation of pain signaling and modulation of neurotransmitter release, attenuation of stress response, and reversal of stress induced analgesia [73].

There are some evidences showing that NOP may play a role in the pathophysiology of IBD suggesting a possible clinical application for compounds targeting these receptors. Sobczak et al. [73] reported that NOP mRNA expression is decreased in human biopsies collected from CD patients in comparison to healthy volunteers. Furthermore, they assessed the antinociceptive effect of SCH 221510-a synthetic NOP agonist, during inflammation in mice and observed a significant decrease in the number of abdominal pain responses in a well-established model of abdominal pain, indicating a potent antinociceptive action [73]. Whether these observations could be translated into human conditions is not known. Nevertheless, all experimental studies on GI inflammatory models report alterations of nociceptin system suggesting its implication in IBD pathology [71].

Opioids have been used as analgesic agents for decades and still attract attention of researchers who look for novel antinociceptive drugs. μ-Opioid receptors (MOR), a well-known target for analgesics, are expressed in myenteric and submucosal plexuses of the gut as well as muscular and immune cells. The localization of opioid receptors is closely related to the role of their ligands in the GI tract, which act as potent antinociceptive and immunomodulatory agents [66]. However, the potential clinical use of opioids is strongly hampered by multiple adverse effects, including CNS-related euphoria and addiction that they cause. Currently extensive research is focused on finding novel ligands with fewer side effects preferably restricted to the periphery to be used in the treatment of abdominal pain.

During the last 20 years, a considerable amount of research efforts has been put on the development of opioid peptides and their potential use as drugs. Peptides have high potency, good selectivity, and low toxicity. A downside, on the other hand, is their vulnerability to rapid enzymatic inactivation in the GI tract and serum. To circumvent this problem, novel peptide-based compounds are synthesized to obtain the most favorable, selectivity, efficacy, and resistance to proteolytic degradation. Most frequently used modifications introduced into natural opioid peptides include insertion of unnatural amino acids and covalent or non-covalent constraints, cyclization, and design of peptidomimetic ligands, glycopeptides, and N-terminal amidinationed analogs [66].

Recently, an analgesic action of novel, cyclic morphiceptin (Tyr-Pro-Phe-Pro-NH_2_) analog, P-317 in the GI tract, has been reported by Sobczak et al. [74]. Morphiceptin is a constituent of bovine β-casein with antinociceptive properties which is rapidly inactivated in vivo by proteases [75, 76]. The new peptide P-317, Dmt-c(D-Lys-Phe-D-Pro-Asp)NH_2_ is resistant to enzymatic degradation in vitro and exhibits long-lasting effects in vivo [77]. It was reported that P-317 significantly decreased the number of mustard oil-induced pain-related behaviors in colitic animals after oral administration [74]. It also improved macroscopic score as well as some biochemical parameters of colonic inflammation. In the same paper, the authors reported significant decrease of MOR and κ-opioid receptor (KOR) in UC and CD patients suggesting that activation of these receptors and restoration of proper opioid signaling may be of benefit in IBD.

Not only peptide but also plant-derived opioid-targeting compounds may enter the group of visceral analgesics in the future. It has been reported that a novel analog of salvinorin A (SA)-a diterpene naturally occurring in South American plant *Salvia divinorum*, PR-38, protects against experimental colitis and reduces abdominal pain in mice by interaction with opioid and cannabinoid receptors [78]. PR-38 significantly attenuated abdominal pain in animal models and in colitic mice through MOR. However, its simultaneous anti-inflammatory effects were mediated by cannabinoid type 1 (CB1) receptors. These observations point to the possible crosstalk between the endogenous opioid and cannabinoid systems especially since the in vitro studies indicated that PR-38 does not bind to CB1 sites.

A crosstalk between MOR, KOR, and CB1 receptors has been reported before in the in vivo studies on peripheral analgesia [79–81]. Both opioid and CB receptors belong to the same subfamily of G protein-coupled receptors (GPCR); they share the same intracellular signaling cascades dependent on Gi/o protein and are localized in a close proximity (<100 A°) on the same cells, what also suggests a potential interaction [82]. Thus, it is likely that opioid and CB1 receptors form heterodimeric or perhaps heterooligomeric structures that might be targeted by drugs designed to treat both visceral pain and inflammation as shown in the preclinical conditions. However, to date, no further translational studies have been conducted in this field hence emergence of such drugs on the market is a matter of the long-term perspective.

Another concept which has recently attracted researchers’ attention is the use of polyunsaturated fatty acids (PUFAs) to combat symptoms of IBD. There are two main groups of biologically important long-chain PUFAs: n-6 PUFA with their first double bond at C6, counting from the methyl C, and n-3 PUFAs with first unsaturated bond at C3 [83]. Both n-3 and n-6 PUFAs are converted to prostanoids, such as prostaglandins and prostacyclins. Moreover, n-3 PUFAs are metabolized by lipoxygenase to protectins, marensins, and resolvins. Although preclinical studies have shown promising results (see details in Michalak et al.) [83], translation of PUFA-based interventions into human conditions appears troublesome. First, it has been shown that total dietary intake of PUFAs positively correlates with the risk of UC [84]. On the other hand, a more detailed big cohort study demonstrated that total dietary n-3 PUFAs, particularly EPA and DHA, protected from UC development in patients older than 45 years [85]. Unfortunately, clinical studies assessing PUFAs therapeutic activity in IBD focus mainly on induction or prolongation of remission, rarely evaluating their analgesic effects. One randomized, controlled trial was performed using seal oil (10 ml, 3x daily) which showed that such treatment reduced disease activity index, normalized n-3/n-6 PUFAs ratio, and alleviated joint-related pain in IBD patients [86]. Besides that report, there is no strong evidence supporting supplementation with n-3 and/or n-6 PUFAs in order to treat pain in IBD and further studies in this field are needed.

Transient receptor potential (TRP) channels are widely distributed in the CNS and peripheral nervous system, including the GI tract and immune cells. Activation or inhibition of selected TRP channels may affect the development of inflammation during IBD, as well as pain signaling. A growing body of evidence indicates that TRPV1 possess anti-inflammatory activity, as stimulation of these TRPs decreases the production of pro-inflammatory cytokines, for example, TNFa and IL-6 [87]. Of note, it has been shown that concomitant blockade of TRPV1 and TRPA1 decreases visceromotor responses at high distension pressures during TNBS-induced colitis more efficiently that blockade of either TRPV1 or TRPA1 alone [88]. This suggests that TRPV1/TRPA1 mixed antagonists may play a role in the intestinal inflammation-induced visceral hyperalgesia. Moreover, stimulation of TRPV4 activates NF-kB, the most important transcription factor during inflammation. Therefore, TRPV4 antagonists may by a promising IBD therapy. It was recently evidenced by Fichna et al. [89] that TRPV4 antagonism alleviates colitis and abdominal pain, paving a pathway to future anti-inflammatory drugs. TRP agonists or antagonists, depending on TRP type, may thus constitute an attractive target in IBD treatment and need further attention [87].

One of the most promising emerging treatment strategies for IBD is inhibition of Janus kinases (JAK1, JAK2, JAK3) and tyrosine kinase 2. JAKs are located at the cytoplasmic tail of cytokine receptors and are activated by receptor-ligand interactions. Upon activation, JAKs undergo auto-phosphorylation with simultaneous phosphorylation of cytokine receptor chains located in their vicinity. These modifications induce conformational changes that allow for binding of one or more signal transducer and activator of transcription (STAT) proteins [90]. Activated STAT molecules act as epigenetic modifiers of gene expression—translocate to the nucleus and facilitate transcription of genes that code various cytokines. The types of JAK activated upon cytokine binding depend on the composition of the receptor with which they are bound. Therefore, the combination of the receptor and JAK types determines which of the seven families of STAT proteins are activated. Consequently, various JAK-STAT activation patterns are observed in reaction to different cytokines. Nevertheless, majority of these cytokines is crucial for lymphocyte activation, function, and proliferation. Thus, inhibition of JAKs results in significant downregulation of cytokine signaling in immune cells which infiltrate intestinal mucosa and contribute to the tissue damage and development of IBD. Recently, a number of small-molecule inhibitors of JAKs have been developed and tested in various models of inflammation. One of them, tofacitinib (CP-690,550), is an oral inhibitor of JAK1, 2, and 3 with in vitro functional specificity for type 1 and 3 over 2, which blocks signaling involving Îł chain-containing cytokines including interleukins 2, 4, 7, 9, 15, and 21 [91]. Good outcomes of preclinical experiments led to human studies on this compound in UC patients. A double-blind, placebo-controlled, phase 2 trial (n=194) showed significant, dose-dependent anti-inflammatory effect as compared to placebo [91]. Unfortunately, the authors did not report the effect of the treatment on abdominal pain score, but global assessment of disease severity according to Mayo scoring system (Funded by Pfizer; ClinicalTrials.gov number, NCT00787202). These encouraging outcomes lead to the funding of three phase 3 trials, two investigating tofacitinib as induction therapy (OCTAVE Induction 1 and 2) and one investigating tofacitinib as maintenance therapy (OCTAVE Sustain) for UC (funded by Pfizer; ClinicalTrials.gov numbers, NCT01465763, NCT01458951, and NCT01458574, respectively) [92]. All trials showed that in patients with moderately to severely active UC, tofacitinib was more effective as induction and maintenance therapy than placebo. Treatment with tofacitinib was associated with increased overall risk of infection compared to placebo and with increased levels of certain lipids without increased risk of cardiovascular events [92]. Recently, Panes et al. [93] evaluated health-related quality of life in UC patients treated with tofacitinib in all mentioned phase 3 studies and reported the impact of this drug on abdominal pain perception. According to the Short-Form-36v2® Health Survey scoring system, patients reported significant relief in abdominal pain in both induction and sustain trials. These results encourage the use of JAK inhibitors in the future treatment of abdominal pain in UC especially since it exhibited a long-lasting effect-52 week in the OCTAVE Sustain study. Phase 4 trials are likely to finish soon; hence, it is expected that first-generation JAK inhibitors will enter the market in the near future (Table 2).

**Table 2:** ᅟ

New concepts in IBD pain alleviation	
NOP targeting	• Antinociceptive effect of synthetic NOP agonist (SCH 221510) • Decrease in the number of abdominal pain responses
New opioid ligands	• Fewer side effects • Peripheral localization
Opioid peptide-based compounds	• Good selectivity, high potency, low toxicity • More resistant to rapid inactivation and proteolytic degradation P-317: decrease in the number of pain-related behaviors, improvement in biochemical parameters of inflammation
Plant-derived opioid receptor-targeting compounds	• Interaction with opioid and cannabinoid receptors PR-38: reduction/attenuation of abdominal pain in mice
JAK inhibitors	• Downregulation of cytokine signaling, anti-inflammatory effect • Impact on abdominal pain perception-significant relief in abdominal pain confirmed in phase 3 studies

*NOPs* nociceptin receptors, *JAKs* Janus kinases (JAK 1, 2, and 3)

## Conclusions

Currently, there is no one standardized method of managing chronic visceral pain in IBD. Traditional treatment relies on multimodal pharmacotherapy that is not specific for IBD only, but used also in other chronic pain conditions. That leads to augmentation of prescribed medication but also increased use of OTC drugs, resulting in adverse effects and analgetic drug dependency, specifically related to opiate use which has been implicated in increased mortality in IBD populations. Abdominal and extra-abdominal pain can also be a cause of lack of compliance between attending physician and a patient, who may feel this most severe symptom remains neglected. Patients tend to present catastrophizing and help-seeking behaviors and may use help of several professionals; therefore, gastroenterologist in charge may not be aware of the intake of additional medication prescribed by colleague. Moreover, to help ease their symptoms, patients may supplement conventional therapies with complementary and alternative medicine, i.e., diverse medical and healthcare systems, practices, and products that are not presently considered part of conventional medicine. Scientific evidence exists only regarding some therapies; for the most part, well-designed scientific studies concerning safety and efficacy of complementary and alternative therapies have not been conducted. The danger lays especially with alternative therapies, which imply replacing the treatment administered by the physician. Therefore, the patient needs to be informed to use such means only as a complement to prescribed medications.

In times of pain, exacerbation patients are forced to use medical leaves, what increases frustration and risk of anxiety and depression, especially considering the fact that these two entities are comorbid with UC and CD. What is more, this constitutes a significant economical burden on both patients and the state. Pain negatively influences patients’ quality of life, regardless of disease activity or subtype.

Ideally, antinociceptive agents would combine safety and efficacy. Development of new drugs and treatment strategies needs to consider not only initial cure rates but also the risk and prevention of pain recurrence. Further research on the management of chronic IBD-related pain is urgently needed since randomized-controlled trials to guide the optimal chronic pain treatment strategy are lacking, especially considering the growing population of IBD sufferers.

Development of novel analgesics for IBD therapy focuses primarily on finding new molecular targets and simultaneously acting on several targets, not only to alleviate pain but also to reduce inflammation what prolongs the therapeutic effect. Alternative approach could base on alterations of nociceptin system, as it is suggested its involvement in IBD pathophysiology and in the regulation of pain signaling and modulation and experiments on mice showed a significant decrease in the number of abdominal pain responses. Human research is needed to establish whether this approach could be translated into human conditions.

In conclusion, pain-related disability has major effects on QoL and on functional and social outcomes in IBD patients. Regular analgesic use is widespread among this group. The focus of current models should be to prevent analgesic dependence and to put forward novel molecules with higher efficacy and improved safety profile.
